# Supplementary material for: Time-series transcriptome analysis identified differentially expressed genes in broiler chicken infected with mixed Eimeria species
Source: Front Genet. 2022 Aug 8;13:886781. doi: 10.3389/fgene.2022.886781 (PMC9393255; doi:10.3389/fgene.2022.886781)
Supplement: Supplementary file 2 [file DataSheet1.ZIP › 7dpi_KEGG.Gsea.1625115961950/KEGG_METABOLISM_OF_XENOBIOTICS_BY_CYTOCHROME_P450.html]

Details for gene set KEGG\_METABOLISM\_OF\_XENOBIOTICS\_BY\_CYTOCHROME\_P450[GSEA]

|  || Dataset | TMM\_7dpi\_gct\_format\_7dpi\_gct\_format.Class\_4dpi.cls #PC\_versus\_NC.Class\_4dpi.cls #PC\_versus\_NC\_repos |
| Phenotype | Class\_4dpi.cls#PC\_versus\_NC\_repos |
| Upregulated in class | 1 |
| GeneSet | KEGG\_METABOLISM\_OF\_XENOBIOTICS\_BY\_CYTOCHROME\_P450 |
| Enrichment Score (ES) | 0.46025294 |
| Normalized Enrichment Score (NES) | 1.7207645 |
| Nominal p-value | 0.011415525 |
| FDR q-value | 0.041851357 |
| FWER p-Value | 0.258 |
Table: GSEA Results Summary

  

Fig 1: Enrichment plot: KEGG\_METABOLISM\_OF\_XENOBIOTICS\_BY\_CYTOCHROME\_P450      
 Profile of the Running ES Score & Positions of GeneSet Members on the Rank Ordered List

  

| SYMBOL | TITLE | RANK IN GENE LIST | RANK METRIC SCORE | RUNNING ES | CORE ENRICHMENT || 1 | GSTA3 | na | 22 | 1.645 | 0.0916 | Yes |
| 2 | MGST1 | na | 67 | 1.378 | 0.1662 | Yes |
| 3 | MGST3 | na | 120 | 1.205 | 0.2302 | Yes |
| 4 | GSTA2 | na | 139 | 1.157 | 0.2944 | Yes |
| 5 | GSTZ1 | na | 161 | 1.120 | 0.3562 | Yes |
| 6 | GSTT1 | na | 164 | 1.112 | 0.4192 | Yes |
| 7 | CYP2C18 | na | 638 | 0.669 | 0.4178 | Yes |
| 8 | UGT1A1 | na | 772 | 0.599 | 0.4407 | Yes |
| 9 | ADH1C | na | 914 | 0.551 | 0.4603 | Yes |
| 10 | GSTO2 | na | 1249 | 0.441 | 0.4575 | No |
| 11 | GSTK1 | na | 1770 | 0.314 | 0.4319 | No |
| 12 | GSTA4 | na | 1872 | 0.294 | 0.4402 | No |
| 13 | DHDH | na | 4357 | -0.036 | 0.2350 | No |
| 14 | ALDH3B2 | na | 4510 | -0.051 | 0.2252 | No |
| 15 | MGST2 | na | 5769 | -0.177 | 0.1303 | No |
| 16 | GSTM2 | na | 6008 | -0.202 | 0.1219 | No |
| 17 | ADH6 | na | 6644 | -0.269 | 0.0843 | No |
| 18 | EPHX1 | na | 6712 | -0.276 | 0.0943 | No |
| 19 | UGT2A1 | na | 7912 | -0.410 | 0.0176 | No |
| 20 | CYP3A4 | na | 9734 | -0.665 | -0.0966 | No |
| 21 | CYP3A5 | na | 9804 | -0.675 | -0.0640 | No |
| 22 | ADH4 | na | 9849 | -0.682 | -0.0290 | No |
| 23 | ALDH1A3 | na | 11961 | -1.725 | -0.1071 | No |
| 24 | CYP1B1 | na | 11987 | -1.957 | 0.0019 | No |
Table: GSEA details [plain text format]

  

Fig 2: KEGG\_METABOLISM\_OF\_XENOBIOTICS\_BY\_CYTOCHROME\_P450      
 Blue-Pink O' Gram in the Space of the Analyzed GeneSet

  

Fig 3: KEGG\_METABOLISM\_OF\_XENOBIOTICS\_BY\_CYTOCHROME\_P450: Random ES distribution      
 Gene set null distribution of ES for **KEGG\_METABOLISM\_OF\_XENOBIOTICS\_BY\_CYTOCHROME\_P450**
